# Genome-wide identification of genes critical for *in vivo* fitness of multi-drug resistant porcine extraintestinal pathogenic *Escherichia coli* by transposon-directed insertion site sequencing using a mouse infection model

**DOI:** 10.1080/21505594.2022.2158708

**Published:** 2023-01-04

**Authors:** Fan Yin, Yan Hu, Zixuan Bu, Yuying Liu, Hui Zhang, Yawen Hu, Ying Xue, Shaowen Li, Chen Tan, Xiabing Chen, Lu Li, Rui Zhou, Qi Huang

**Affiliations:** a State Key Laboratory of Agricultural Microbiology, College of Veterinary Medicine, Huazhong Agricultural University, Wuhan, China; b College of Animal Sciences & Technology, Huazhong Agricultural University, Wuhan, China; c Cooperative Innovation Center for Sustainable Pig Production, College of Veterinary Medicine, Huazhong Agricultural University, Wuhan, China; d Ministry of Science and Technology, International Research Center for Animal Disease, Wuhan, China; e Institute of Animal Husbandry and Veterinary Science, Wuhan Academy of Agricultural Science and Technology, Wuhan, China; f The HZAU-HVSEN Institute, Wuhan, China

**Keywords:** Transposon mutagenesis, transposon-directed insertion site sequencing (TraDIS), genome-wide screening, extraintestinal pathogenic *Escherichia coli*, fitness factor

## Abstract

Extraintestinal pathogenic *Escherichia coli* (ExPEC) is an important zoonotic pathogen. Recently, ExPEC has been reported to be an emerging problem in pig farming. However, the mechanism of pathogenicity of porcine ExPEC remains to be revealed. In this study, we constructed a transposon (Tn) mutagenesis library covering Tn insertion in over 72% of the chromosome-encoded genes of a virulent and multi-drug resistant porcine ExPEC strain PCN033. By using a mouse infection model, a transposon-directed insertion site sequencing (TraDIS) assay was performed to identify *in vivo* fitness factors. By comparing the Tn insertion frequencies between the input Tn library and the recovered library from different organs, 64 genes were identified to be involved in fitness during systemic infection. 15 genes were selected and individual gene deletion mutants were constructed. The *in vivo* fitness was evaluated by using a competitive infection assay. Among them, Δ*fimG* was significantly outcompeted by the WT strain *in vivo* and showed defective adhesion to host cells. *rfa* which was involved in lipopolysaccharide biosynthesis was shown to be critical for *in vivo* fitness which may have resulted from its role in the resistance to serum killing. In addition, several metabolic genes including *fepB*, *sdhC*, *fepG*, *gltS*, *dcuA*, *ccmH*, *ddpD*, *narU*, *glpD*, *malM*, and *yabL* and two regulatory genes *metJ* and *baeS* were shown as important determinants of *in vivo* fitness of porcine ExPEC. Collectively, this study performed a genome-wide screening for *in vivo* fitness factors which will be important for understanding the pathogenicity of porcine ExPEC.

## Introduction

Extraintestinal pathogenic *Escherichia coli* (ExPEC) is posing an important threat to public health, which is involved in infections or even death of both humans and farm animals [1]. Differing from commensal or intestinal pathogenic *E. coli*, ExPEC causes a wide range of extraintestinal infections which can colonize the intestine asymptomatically as the reservoir of infection [2,3]. In human, ExPEC cause diseases including urinary tract infections (UTIs), neonatal meningitis, and septicemia [4]. It is estimated that ExPEC accounts for more than 60% of human UTIs and 27% of documented bacteremia which causes significant public health problems and economic burdens [5–7]. ExPEC is also a common bacterial pathogen in farm animals. Apart from the well-characterized avian pathogenic *E. coli* (APEC) that can cause multiple local and systemic infections in poultry which is one of the leading causes of morbidity and mortality [8], ExPEC in pigs has recently emerged as a fatal pathogen causing porcine meningitis, pneumonia, and septicemia, resulting in huge economic losses [9–11]. The incidence of ExPEC in pigs is reported to be increasing in recent years [11–13]. In addition, ExPEC of animal origins has been recognized as an important source of foodborne illness [14]. The high level of antimicrobial resistance and high flexibility in genome diversity brings enormous difficulties in controlling and preventing porcine ExPEC infections.

Understanding the mechanism of bacterial pathogenesis is critical for the development of drugs and vaccines. *E. coli* populations have high genetic and phenotypic diversity [15]. Different types of ExPEC carry specific fitness factors and virulence factors (VFs) to adapt to various niches [16]. Uropathogenic *E. coli* (UPEC) produce P ﬁmbriae to facilitate adherence to renal cells [17,18] and are equipped with a higher amino acid biosynthetic capacity which is important for growth in urine [19]. Neonatal meningitis *E. coli* (NMEC) and sepsis‑associated *E. coli* (SEPEC) strains have the ability to survive in the bloodstream in which their important VFs include capsular antigens to protect against phagocytosis, Iss protein for survival in serum, and IbeABC to promote host cells invasion [4,20,21]. APEC carry avian-associated ColV plasmids essential for poultry adaptation that encompass virulence genes such as *iucC*, *iucD*, *iutA*, *cvaA*, *etsA*, *hlyF*, *ompT*, *cvaB*, *cvaC*, and *cvi* [22,23]. Nevertheless, some VFs such as the iron acquisition factors and some adhesins are shared among different pathotypes of ExPEC [4]. Moreover, efforts have been made to analyze the population structure of ExPEC by using comparative genomics approaches. However, the results suggest that it is still difficult to reveal the underlying mechanism of pathogenesis and identify disease-specific functional or genetic coordinates of ExPEC [24,25].

Porcine ExPEC has recently become an emerging problem in pig farming in China [10,26,27]. Increasing incidence and high levels of antimicrobial resistance make porcine ExPEC a major bacterial pathogen in the pig industry [11,28]. However, the mechanisms of pathogenicity are less understood. So far, the type VI secretion system, Rhs proteins, the twin-arginine translocation system, the biosynthesis of nucleotide and extracellular polysaccharides, and the global regulators Fur and FNR have been reported critical for the virulence of porcine ExPEC [9,29–31]. However, the underlying mechanism of pathogenesis is far from comprehensively elaborated. Therefore, further identification of factors involved in pathogenicity is necessary to provide a better understanding of this pathogen.

Transposon-directed insertion site sequencing (TraDIS) has become a powerful approach that combines transposon mutagenesis with high-throughput sequencing to do genome-wide screenings [32–34]. By comparing the transposon insertion frequency between the treated and untreated transposon libraries, TraDIS allows the identification of a large number of functional genes in a single experiment. It has been used to identify essential genes, drug-resistant genes, and *in vivo* fitness genes in several important bacterial pathogens [35–38].

Our previous studies isolated and characterized a number of clinical ExPEC strains from pigs and identified a strain PCN033 which is multi-drug resistant and virulent, belonging to sequence type 597, serogroup O11, and phylogenetic group D [26,39,40]. Using a pig infection model, it was demonstrated that the PCN033 strain possessed high virulence which can cause systemic infection including neurological symptoms, bacteremia, and even death [27]. The high virulence of the PCN033 strain was also observed in the mouse infection model where the mice infected with this strain also exhibited similar symptoms to those in the pig infection model, suggesting that the mouse infection model can be a surrogate model of pig infection [31,41]. In this study, we constructed a transposon mutagenesis library of ExPEC PCN033 strain covering Tn insertion in over 72% of the chromosome-encoded genes and performed a TraDIS assay using a mouse infection model which identified a variety of genes potentially involved in *in vivo* fitness during infection. By constructing individual gene deletion mutants, we further confirmed that genes involved in fimbriae and LPS biosynthesis, metabolism, and transcription regulation are critical for porcine ExPEC to establish systemic infection. Our genome-wide screening for *in vivo* fitness factors will be important for understanding the pathogenesis of porcine ExPEC.

## Results

### Transposon mutagenesis library construction and evaluation

**Figure 1.**
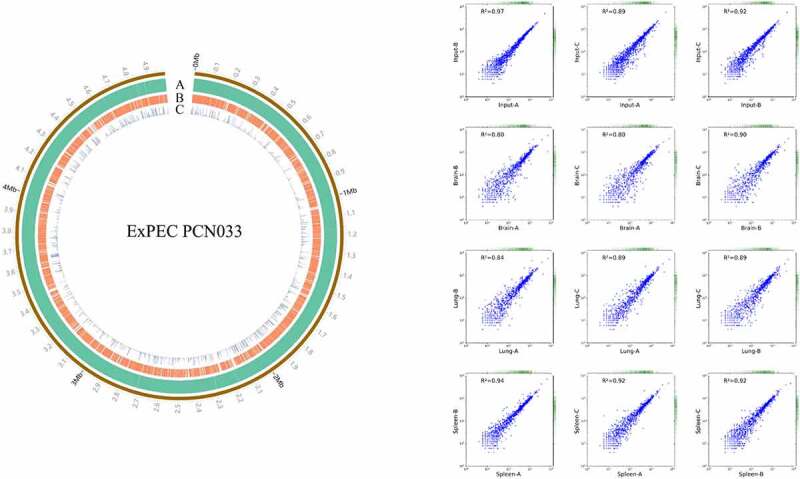
Distribution of transposon insertion sites and correlation coefficients. (a) distribution of transposon insertion sites of the input library. the transposon insertion sites were mapped to the genome of ExPEC strain PCN033 and the data was visualized by using circos. Circle a represents the genome of ExPEC PCN033 strain. Circle B shows the transposon insertion sites. Circle C shows the mapped reads for each insertion site. The figure showed the data of one of the input libraries. (b) correlation coefficients between duplicates of input and output libraries. the correlation coefficients were calculated using the StandardScaler submodule of the sklearn module of the python programming language. DEseq2 was used to compare the change in the total number of insertion reads for each gene between the input and output libraries. Fold-change (FC) > 2 and *p* value <0.05 was used as the threshold to identify enriched genes.

The ExPEC PCN033 strain is a virulent clinical isolate and a multi-drug resistant strain [39]. So, we designed and constructed a plasmid pSAM-Tat-Apra for transposon mutagenesis for this strain (Fig. S1A). Transposon mutagenesis was successfully achieved as verified by PCR amplification and insertion site identification. Randomly selected transposon mutants showed transposition at different sites on the chromosome (Fig. S1B). A transposon library containing approximately 68,000 individual transposon mutants was constructed. By sequencing the library and mapping the transposon insertion sites to the genome, it was shown that a total of 3541 genes contained insertions, comprising about 72% of the total chromosome-encoded genes (Figure 1(a)).

### Identification of genes critical for *in vivo* fitness by TraDIS

**Figure 2.**
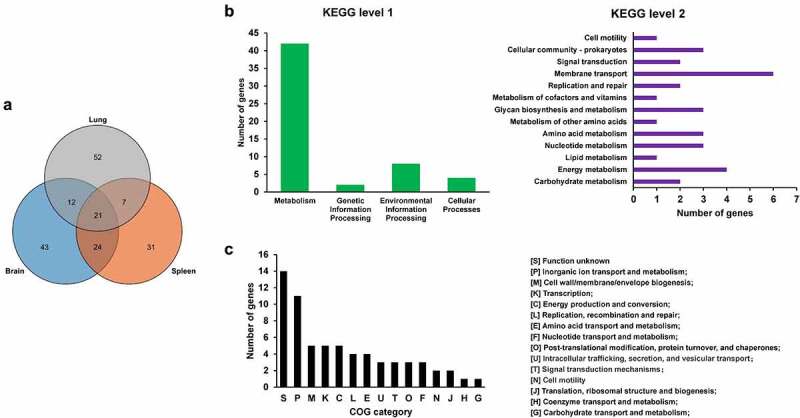
Genes essential for *in vivo* fitness. (a) venn diagram of the fitness genes identified in different organs. the diagram was generated by using Tbtools according to Table S3. (b) KEGG pathway enrichment of the fitness genes. the fitness genes were analyzed by using BlastKOALA tool (https://www.Kegg.jp/blastkoala/) to assign K numbers which were then used for pathway enrichment by using KEGG mapper (https://www.Genome.jp/kegg/mapper/search.Html). The numbers inside the vertical bars are the total number of genes belonging to each level 2 KEGG pathway. (c) COG enrichment of the fitness genes. the fitness genes were analyzed using the EggNOG v5.0 database (http://eggnog5.Embl.de/#/app/home).

To screen for genes critical for *in vivo* fitness, the transposon library was used to infect mice intraperitoneally. The input library and the bacteria recovered from the organs including the brain, spleen, and lung (output libraries) were isolated and sequenced. By calculating the insertion frequency between the input and output libraries, genes with differential transposon insertion were identified. As shown in Figure 1(b), the correlation coefficients of the genes with differential insertion frequency were between 0.8–0.97, indicating consistency between biological replicates. A total of 190 genes were identified with differential insertions (FC > 2, *p* value <0.05) in the output libraries recovered from brain, spleen, and lung tissues compared with the input library, which may be potentially involved in *in vivo* fitness of ExPEC (Figure 2 and Table S3). Among them, 64 genes were identified that were involved in the fitness in at least two organs (Figure 2 and Table S3). KEGG pathway enrichment analysis with these 64 genes revealed that most of the genes are involved in metabolism, among which membrane transport and energy metabolism were the most enriched level 2 KEGG pathways. By COG enrichment, most of the TraDIS-identified genes belonged to the categories including functional unknown [S], inorganic ion transport and metabolism [P], Cell wall/membrane/envelope biogenesis [M], Transcription [K], and Energy production and conversion [C].

### *fimG* is required for adhesion of ExPEC

**Figure 3.**
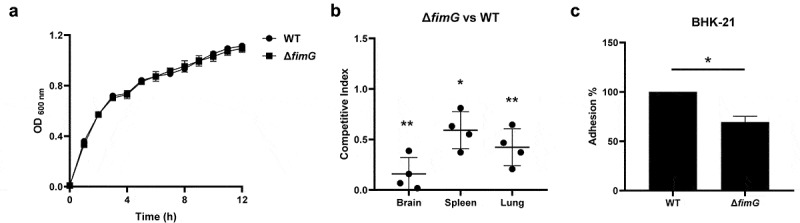
*fimG* is required for adhesion. (a) growth assay. Cells of WT and Δ*fimG* strains were inoculated 1:100 from overnight-grown culture to LB. The growth was monitored using an automatic plate reader (bioscreen C, FP-1100-C, oy growth curves AB, USA) at 37°C with shaking. Five replicates were tested for each strain. The data shown are the mean ± standard error of the mean (SEM). (b) competitive infection assay. 200 μL of cell suspension containing a total of 6 × 10^5^ CFU of WT and Δ*fimG* cells with a ratio of 1:1 was used to intraperitoneally inject mice with five mice in each group. At 12 hpi, mice were euthanized, and brain, spleen, and lung tissues were taken, homogenized, diluted, and plated on LB agar plates with and without appropriate antibiotics, respectively, to distinguish the mutant and the wild-type cells. The bacteria were enumerated and the competition index (CI) was calculated as defined as the mutant-to-WT ratio within the recovered sample, divided by the corresponding ratio in the inoculum. The student’s t test (two-tail, unpaired) was used to calculate the statistical difference between the mean CI value and 1. * indicates *p* <0.05 and ** indicates *p* <0.01. (c) cell adhesion assay. the bacterial cells at the mid-log phase were used to infect BHK-21 cells with a multiplicity of infection (MOI) of 10:1 in a six-well plate followed by incubation at 37°C for 1 h. The mixture was washed with sterile PBS and then sterile water was added to the mixture which was incubated at 4°C for 1 h for cell lysis. The lysate was serially diluted and applied to LB agar plates for bacterial counting. The student’s t test (two-tail, unpaired) was used to calculate the statistical difference between the two groups. * indicates *p* <0.05.

*fimG* encodes the minor component of type 1 fimbriae, which has been identified with a decreased transposon insertion in the output libraries recovered from all three organs (Table S3). To further confirm its role in pathogenesis, we constructed the deletion mutant of *fimG*. The growth curve data showed that the mutant did not show growth defects compared with WT strain (Figure 3(a)). Competitive animal infection assay, a sensitive and accurate approach to determine the degree of virulence attenuation [42], was used to evaluate the *in vivo* colonization of the mutant. A control assay using ExPEC PCN033 strain and PCN033-Chl strain which was the PCN033 strain inserted with a chloramphenicol resistance cassette in the intergenic region of the chromosome, to inject mice simultaneously. The result showed that the insertion of the resistance cassette did not interfere with the competitive index (Figure S2). By performing a competitive animal infection assay using Δ*fimG* and WT strain, it was shown that the mutant strain displayed significantly reduced colonization in the brain (CI = 0.158), spleen (CI = 0.591), and lung (CI = 0.338) compared with the WT strain (Figure 3(b)). Then, *in vitro* cell adhesion assay was carried out which showed that the Δ*fimG* strain displayed a significantly lower ability to adhere to and be internalized by BHK-21 cells (Figure 3(c)). Therefore, our results showed that *fimG* is critical for host adhesion of ExPEC.

### Lipopolysaccharide biosynthesis is critical for serum survival

**Figure 4.**
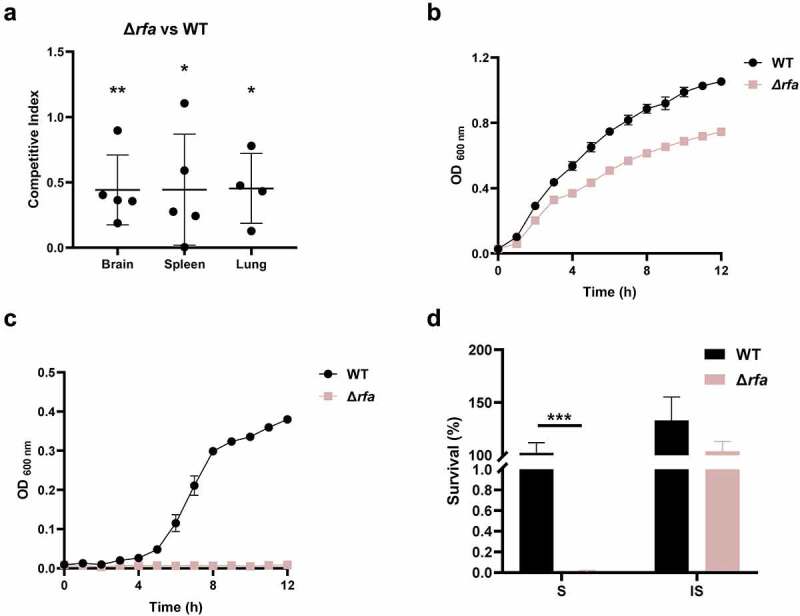
*Rfa* is critical for serum survival. (a) competitive infection assay. 200 μL of cell suspension containing a total of 6 × 10^5^ CFU of WT and Δ*rfa* cells with a ratio of 1:1 was used to intraperitoneally inject mice with five mice in each group. At 12 hpi, mice were euthanized, and brain, spleen, and lung tissues were taken, homogenized, diluted, and plated on LB agar plates with and without appropriate antibiotics, respectively, to distinguish the mutant and the wild-type cells. The bacteria were enumerated and the competition was calculated. The student’s t test (two-tail, unpaired) was used to calculate the statistical difference between the mean CI value and 1. * indicates *p* <0.05, and ** indicates *p* <0.01. (b and c) growth assay. Cells of WT and Δ*rfa* strains were inoculated 1:100 from overnight-grown culture to LB (A) or supplemented with 75% fresh mouse serum (B). The growth was monitored using an automatic plate reader (bioscreen C, FP-1100-C, oy growth curves AB, USA) at 37°C with shaking. Five replicates were tested for each strain. The data shown are the mean ± standard error of the mean (SEM). (d) serum killing assay. Cells of WT and Δ*rfa* strains were subcultured in LB from overnight-grown culture and grown at 37°C to the mid-log phase. The cells were washed with sterile saline and 5 × 10^5^ CFU of the cells was incubated with 75% fresh (S) or heat-inactivated serum (IS) for 20 min at 37°C. Afterward, the mixture was serially diluted and plated on LB agar plates for viable cell enumeration. The assay was performed in triplicates and the data shown are the mean ± standard error of the mean (SEM). The student’s t test (two-tail, unpaired) was used to calculate the statistical difference between the two groups. *** indicates *p* <0.001.

*rfaI*, *rfaJ*, and *rfaL* (also known as *waaL*) are within the same operon involved in the biosynthesis of lipopolysaccharide. These three genes have been identified in the TraDIS assay (Table S3). A mutant strain lacking the *rfa* operon was constructed (Δ*rfa*). The competitive infection assay confirmed that Δ*rfa* strain showed a reduced ability to colonize in mice (Figure 4(a)). The growth assay revealed that Δ*rfa* showed slightly reduced growth in LB medium compared with the WT strain in which the maximum growth rate and the total growth yield of the mutant were 68.1% and 70.4%, respectively, compared to that of the WT strain (Figure 4(b)). In contrast, the growth of Δ*rfa* was almost abolished in the presence of 75% fresh mice serum (Figure 4(c)). This was consistent with the serum killing assay results demonstrating that the Δ*rfa* strain was markedly more susceptible to fresh serum (S) but not the heat-inactivated serum (IS) than the WT strain (Figure 4(d)). Therefore, the data suggest that *rfa* operon plays an important role in fitness *in vivo* which could be mediated by its effects on resistance to serum killing.

### Several metabolic genes are involved in colonization *in vivo*

**Figure 5.**
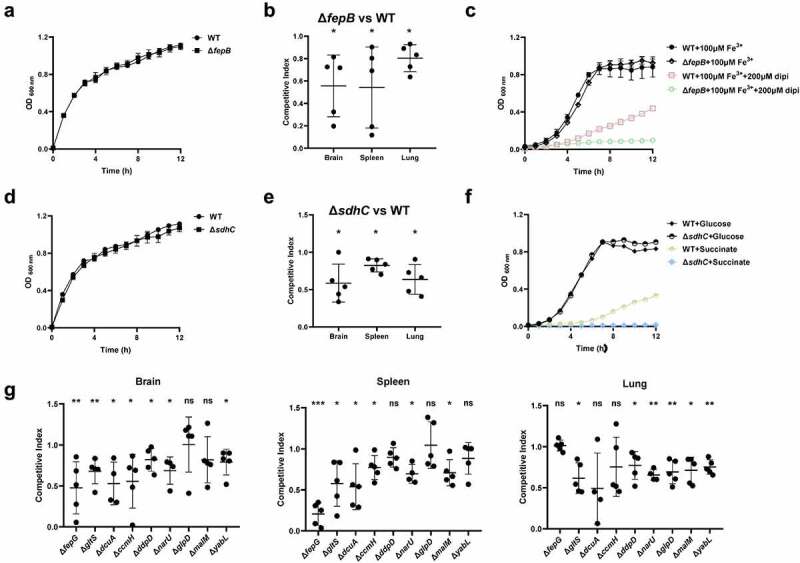
Fitness of metabolism-related mutants. (a, c, d, and f) growth assay. Cells of each indicated strain were inoculated 1:100 from overnight-grown culture to LB (a and e), M9 medium or M9 medium supplemented with the additives (D and G). The growth was monitored using an automatic plate reader (bioscreen C, FP-1100-C, oy growth curves AB, USA) at 37°C with shaking. Five replicates were tested for each strain. The data shown are the mean ± standard error of the mean (SEM). (b, e, and g) competitive infection assay. 200 μL of cell suspension containing a total of 6 × 10^5^ CFU of cells of WT and each indicated strain with a ratio of 1:1 was used to intraperitoneally inject mice with five mice in each group. At 12 hpi, mice were euthanized, and brain, spleen, and lung tissues were taken, homogenized, diluted, and plated on LB agar plates with and without appropriate antibiotics, respectively, to distinguish the mutant and the wild-type cells. The bacteria were enumerated and the competition index (CI) was calculated. The student’s t test (two-tail, unpaired) was used to calculate the statistical difference between the mean CI value and 1. ns indicates no significant difference, * indicates *p* <0.05, ** indicates *p* <0.01, *** indicates *p* <0.001.

**Figure 6.**
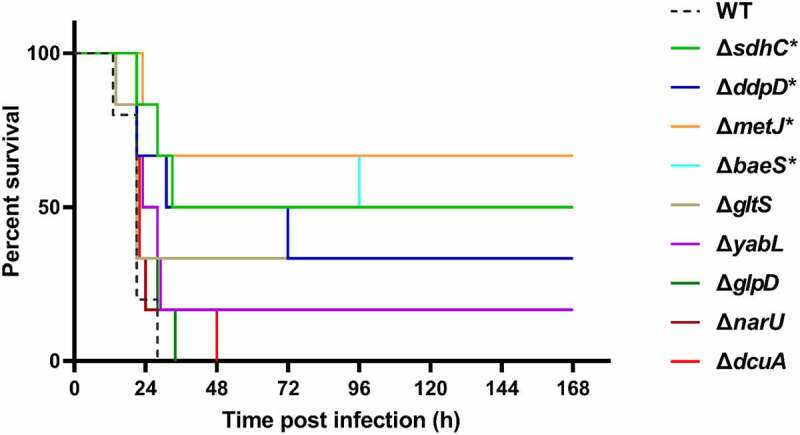
Mice survival assay. the indicated ExPEC strains were subcultured from overnight-grown cultures into LB and grown to the mid-log phase at 37°C with shaking. The cells were harvested by centrifugation, washed with sterile saline, and diluted to appropriate concentrations with saline. 0.2 ml of the bacteria suspension containing approximately 6.9 × 10^5^ CFU was used to infect 4-week-old Kunming mice intraperitoneally with 6 mice in each group (except the WT group which harbored 5 mice). The survival of mice was recorded. Statistical differences in survival between the mutant group and the WT group were determined using the log-rank test. * indicates *p* <0.05.

In the TraDIS results, a large proportion of genes related to bacterial metabolism were identified to be required for fitness *in vivo* (Figure 2(b)). *fepB* is part of the ABC transporter complex FepBDGC involved in ferric enterobactin uptake [43]. To investigate the role of *fepB* in the pathogenesis of ExPEC, we constructed a *fepB* mutant strain. Animal infection assay revealed that the ability of the mutant to colonize in the brain, spleen, and lung tissues of mice was significantly lower than that of the WT strain, indicating that *fepB* significantly improved ExPEC colonization *in vivo* (Figure 5(b). Growth assay revealed that Δ*fepB* showed a similar growth with WT strain in LB (Figure 5(a)) or in M9 medium supplemented with sufficient iron (Figure 5(c)). However, when iron was depleted by the addition of 2, 2’-bipyridine (dipi), compared with the WT strain that the growth was partially affected, the growth of Δ*fepB* was almost abolished (Figure 5(c)). This suggests that Δ*fepB* is involved in the growth under conditions with limited iron. *sdhC* encodes the membrane-anchored subunit of succinate dehydrogenase, which is involved in the tricarboxylic acid cycle pathway of carbohydrate metabolism, catalyzing succinate to fumarate [44]. It was shown that the growth of the Δ*sdhC* was comparable to that of the WT strain in LB medium (Figure 5(d)). However, the competitive infection assay showed that the deletion of *sdhC* significantly compromised the ability of ExPEC to colonize the brain, spleen, and lung (Figure 5(e)). We further tested the growth of Δ*sdhC* in the M9 medium containing glucose or succinate as the only carbon source. The Δ*sdhC* strain grew at a similar rate to the WT strain in the M9-glucose medium (Figure 5(f)). It was also seen that the WT strain grow slower in the M9- succinate medium than in the M9-glucose medium (Figure 5(f)), which was consistent with the fact that glucose is a preferred carbon source for *E. coli*. However, the growth of Δ*sdhC* strain was almost abolished in the M9-succinate medium while the growth of the WT strain was only partially influenced (Figure 5(f)). In addition, we constructed deletion mutants of *fepG*, *gltS*, *dcuA*, *ccmH*, *ddpD*, *narU*, *glpD*, *malM*, and *yabL*, which are involved in ferric enterobactin transport, glutamate transport, C4-dicarboxylate transport, c-type cytochrome biosynthesis, D, D-dipeptide transport, nitrate/nitrite transport, glycerol metabolism, maltose uptake, and thiamine import, respectively. There was no significant difference in growth in LB medium between the mutants and the WT strain (data not shown). However, the competitive infection assay revealed that their *in vivo* fitness was compromised in at least one organ tested (Figure 5(g)). To further determine the contribution of each gene to the pathogenicity of ExPEC, we tested the survival of mice infected with WT, Δ*sdhC*, Δ*gltS*, Δ*yabL*, Δ*dcuA*, Δ*ddpD*, Δ*narU*, and Δ*glpD* strain, respectively. As shown in Figure 6, the mice infected with Δ*sdhC* or Δ*ddpD* showed significantly higher survival, while the other strains showed comparable virulence to the WT strain.

### Two regulators are identified critical for *in vivo* fitness and virulence

**Figure 7.**
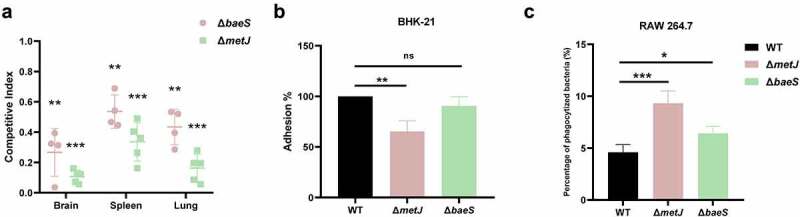
Two regulatory genes are critical fitness factors. (a) competitive infection assay. 200 μL of cell suspension containing a total of 6 × 10^5^ CFU of cells of WT and each indicated strain with a ratio of 1:1 was used to intraperitoneally inject mice with five mice in each group. At 12 hpi, mice were euthanized, and brain, spleen, and lung tissues were taken, homogenized, diluted, and plated on LB agar plates with and without appropriate antibiotics, respectively, to distinguish the mutant and the wild-type cells. The bacteria were enumerated and the competition index (CI) was calculated. The student’s t test (two-tail, unpaired) was used to calculate the statistical difference between the mean CI value and 1. ** indicates *p* <0.01, and *** indicates *p* <0.001. (b) cell adhesion assay. the bacterial cells at the mid-log phase were used to infect BHK-21 cells with a multiplicity of infection (MOI) of 10:1 in a six-well plate followed by incubation at 37°C for 1 h. The mixture was washed with sterile PBS and then sterile water was added to the mixture which was incubated at 4°C for 1 h for cell lysis. The lysate was serially diluted and applied to LB agar plates for bacterial counting. (c) macrophage phagocytosis assay. the bacterial cells were mixed with RAW 264.7 cells with a multiplicity of infection (MOI) of 10:1 followed by incubation at 37°C for 1 h. The cells were then washed twice with sterile PBS, and incubated with PBS containing chloramphenicol (final concentration 50 μg/ml). The macrophage cells were then lysed with sterile water and the lysate was serially diluted and applied to LB agar plates for bacterial counting. The student’s t test (two-tail, unpaired) was used to calculate the statistical difference between the two groups. ns indicates no significant difference, * indicates *p* <0.05, ** indicates *p* <0.01, ** indicates *p* <0.001.

*metJ* which encodes a transcription regulator [45] and *baeS* which encodes the sensor kinase of the two-component system BaeSR [46] were identified in the TraDIS assay. The deletion mutants of *metJ* and *baeS* were constructed. It was shown that both of the deletion mutants were significantly outcompeted by the WT strain in the brain, spleen, and lung, in which Δ*metJ* strain had a very low competitive index indicating a significant colonization defect compared with the WT strain (Figure 7(a)). To further confirm the roles of *metJ* and *baeS* in the pathogenesis of ExPEC, adhesion assay and phagocytosis by macrophages were carried out. It was shown that the deletion of *metJ* rather than *baeS* significantly compromised the adhesion of ExPEC to BHK-21 cells (Figure 7(b)). Also, both of Δ*metJ* and Δ*baeS* were easier to be phagocytized by RAW 264.7 macrophages than WT cells (Figure 7(c)). The survival assay further showed that the virulence of Δ*metJ* and Δ*baeS* was significantly attenuated compared with the WT strain (Figure 6). These results suggest that MetJ and BaeSR two-component system are virulence regulators of ExPEC.

## Discussion

Compared to intestinal pathogenic *E. coli*, ExPEC causes extraintestinal infections including meningitis, pneumonia, or septicemia, leading to more severe consequences. ExPEC not only threatens human health but also causes serious problems in animal husbandry. Recently, ExPEC has been reported to be an emerging problem of pig farming in which its incidence in pigs is increasing and highly virulent strains have been reported [11,27,47]. So far, there are no effective vaccines developed for preventing ExPEC infection in pigs. Moreover, porcine ExPEC isolates have been revealed to possess broad-spectrum antimicrobial resistance, significantly compromising the efficacy of antibiotic treatment [28]. Therefore, there is an urgent need for research to understand the mechanism of the pathogenesis of porcine ExPEC, which is important for the development of novel vaccines and antimicrobials.

The ability to adapt to and survive in adverse environments is critical for bacterial pathogens to establish infection. *In vitro* conditions that mimic the *in vivo* environment have been widely used to study the mechanism of the pathogenesis of bacterial pathogens [48–50]. However, bacteria encounter complicated challenges during infection *in vivo*, especially for ExPEC which causes systemic infection. Therefore, elucidating the mechanism of *in vivo* fitness is important for understanding the pathogenesis of ExPEC. TraDIS has recently become a popular approach that offers the chance to do genome-wide identification of *in vivo* fitness factors in one shot [35,36,51,52]. One of the key steps of performing TraDIS is the construction of the transposon mutagenesis library. Mariner and Tn5 transposons are the most popular ones used for mutagenesis due to convenient manipulations, broad-host range, and more importantly the nature of near-random insertion [35,53]. Various approaches have been utilized to deliver the transposon into the recipient cells, including the phage delivery systems [51], plasmid delivery systems [54,55], and using *in vitro* assembled transposase [37]. In this study, a clinically isolated and multi-drug resistant strain ExPEC PCN033 was used as the recipient strain to generate the transposon mutagenesis library in which mariner transposon encoded by a suicide plasmid was delivered by transconjugation. A Tn library consisting of over 68,000 mutants was generated, which covered 72% of the genes encoded by the genome as revealed by library sequencing and genome mapping (Figure 1(a)), indicating a near-saturating library.

Mouse with intraperitoneal injection was then used to model the infection of ExPEC to identify the *in vivo* fitness genes of the ExPEC PCN033 strain. Several other routes of infection have also been reported for establishing the mouse infection model, including the intravenous, intranasal routes, and oral administration, depending on the type of pathogen [56]. For pathogens that cause systemic infection, such as ExPEC, intraperitoneal injection is a commonly used route of infection when using the mouse model of infection [57,58]. However, it should be noted that ExPEC enters extraintestinal sites and cause systemic infection from the intestine or through the urinary tract system [59]. Therefore, using the natural host pigs as the model of infection will be of value for future pathogenesis studies of porcine ExPEC despite the limitations of pig models including variations in genetic background, health conditions, and high expenses.

In this study, we constructed a Tn library and for the first time performed a TraDIS assay to do genome-wide identification of *in vivo* fitness factors with porcine ExPEC using a mouse infection model. A total of 64 genes were identified as essential fitness factors during systemic infection. Among these genes, *fimG*, encoding the minor component of type 1 fimbriae, was shown to be involved in fitness [60]. By performing cell adhesion assay, we confirmed that *fimG* plays an important role in adhesion to host cells. Type 1 fimbriae have also been reported as an important virulence factor in UPEC [60–62]. *fepG* and *fepB* encode the components of a transporter involved in the uptake of ferric enterobactin, a siderophore important for the virulence of ExPEC [63,64]. They were also identified in our TraDIS assay to be required for *in vivo* fitness of porcine ExPEC. Our *in vitro* assay further demonstrated that *fepB* is required for ferric iron uptake. The Fep system serves as a siderophore import system and allows the uptake of iron through both the enterobactin and salmochelin siderophores [65,66]. Some bacteria employ other siderophores to scavenge iron that do not depend on the Fep system, such as aerobactin [67] and yersiniabactin [68]. It should be noted that ExPEC PCN033 strain does not produce yersiniabactin but contains an aerobactin-encoding gene on the plasmid. Therefore, it remains to be investigated the role of the plasmid-encoded aerobactin synthesis and import system. In addition, we identified several genes involved in the biosynthesis of lipopolysaccharide core (*rfa*) in our TraDIS assay. It was further shown that Δ*rfa* displayed remarkable growth defects in fresh serum but not in heat-inactivated serum, indicating that lipopolysaccharides play important role in protecting ExPEC from being killed by heat-sensitive antimicrobial substances in serum, such as complements. The critical role of LPS biosynthesis in the resistance of serum killing has also been reported by other groups [9,69–71]. This could be attributed to the critical role of the LPS O-antigen in resistance to complement killing and hydrolysis of lysozyme [71,72].

Bacterial metabolism has been recently recognized to play an important role in pathogenesis. By KEGG pathway enrichment analysis, among the 64 genes identified by TraDIS, 42 genes are metabolism-related (Figure 2(b)). Mouse competitive infection assays further validated the involvement of *fepB*, *sdhC*, *fepG*, *gltS*, *dcuA*, *ccmH*, *ddpD*, *narU*, *glpD*, *malM*, and *yabL* in *in vivo* fitness. Consistently, a previous TraDIS study with an ExPEC strain of avian origin also shows that metabolism genes comprise the most enriched group that is essential for *in vivo* fitness [58]. In UPEC, a significant number of proteins exhibited differential expression in response to urine treatment. Among them, metabolic genes including those involved in peptide uptake, gluconeogenesis, and the tricarboxylic acid cycle were demonstrated to be required for fitness during urinary tract infection [73]. Among these genes, *sdhC* which is a subunit of the succinate dehydrogenase catalyzing the production of fumarate from succinate has also been reported to be important for the virulence of other bacterial pathogens, such as enterohemorrhagic *E. coli* [74] and *Salmonella* [75]. Recently, metabolic crosstalk between bacteria and host cells is becoming an emerging research hotspot [76]. Among the critical metabolites, succinate has been shown to modulate host inflammatory response [77]. Whether the increased succinate level in the Δ*sdhC* mutant has an impact on the host innate immune response to facilitate bacteria clearance remains to be revealed. *ddpD* is an uncharacterized putative D, D-dipeptide transporter in *E. coli* in addition to the other reported transporters such as DppA [78], DtpA [79], DtpB [79], DtpC [80], and DtpD [81]. The mouse survival assay further confirmed the critical role of *ddpD* for the virulence of ExPEC (Figure 6). Therefore, its role in the pathogenesis of ExPEC is worth further investigation. It should be noted that although *dcuA*, *gltS*, *narU*, *glpD*, and *yabL* were outcompeted by the WT strain *in vivo*, mouse assay with each individual mutant showed that they had similar overall virulence in terms of mice survival (Figure 6). This could be attributed to the existence of other functional redundant genes. For example, *dcuA* encodes a transporter for C4-dicarboxylate, such as succinate, L-malate, L-tartrate, and L-aspartate. Apart from DcuA, another C4-dicarboxylate transporter DcuB exists in *E. coli*. These two transporters have preferred substrates, and DucA is constitutively expressed while DcuB is expressed only anaerobically [82,83]. *gltS* encodes a glutamate transporter in *E. coli*. Moreover, there are three other glutamate transporters in *E. coli*, among which Glts is responsible for around 25% of the total glutamate transport [84]. In addition to NarU, *E. coli* produce another transporter NarK for nitrate uptake [85]. But, it has been shown that NarU is more important during nutrient starvation or very slow growth [86]. Therefore, disrupting these genes may affect their capability of *in vivo* competition and fitness while may not necessarily decrease the overall virulence.

In addition, a regulatory gene *metJ*, encoding a transcriptional repressor involved in methionine biosynthesis, was identified as critical for *in vivo* fitness of ExPEC. Deleting *metJ* may result in the increase of methionine biosynthesis in ExPEC. It has been reported that increasing exogenous or endogenous methylthioadenosine (MTA), a methionine-derived metabolite, or deletion of *metJ* could suppress the virulence of *Salmonella* [87]. MetJ is also reported to be required for the virulence of other bacteria, including *Vibrio cholera* [88] and *Pectobacterium atrosepticum* [89]. This indicates a critical role of bacterial methionine metabolism for virulence. As methionine metabolism is intimately related to methylation processes, Bourgeois *et al*. investigated the influence of *metJ* deletion on the DNA methylome of *Salmonella* [90]. However, the results show that the methylome is remarkably static even in the *metJ* deletion strain. Therefore, the exact role of *metJ* in the pathogenesis of ExPEC needs further investigation.

A total of 15 genes out of the 64 genes identified from the TraDIS were selected for validation of which the results of the competitive infection assay were summarized in Table 1. They showed different fitness abilities in different organs. Moreover, it was noted that in the TraDIS assay, some genes showed fitness defects in only one of the organs, in which 43 genes were identified only in the brain, 52 genes only in the lung, and 31 genes only in the spleen (Figure 2(a) and Table S3). This may be because different organs have their specific microenvironments as well as immune defense mechanisms [91]. For example, Δ*glpD* only shows colonization defects in the lung but not in the brain and spleen (Table 1), this could be due to the aerobic condition in the lung, and *glpD* is required for glycerol metabolism in aerobic growth [92]. It will be interesting to identify genes critical for local infections in specific organs in the future.

**Table 1.** Summary of competitive index.

Strain	Organ								
	Brain			Spleen			Lung		
	CI mean	*p* value	significance	CI mean	*p* value	significance	CI mean	*p* value	significance
Δ*fimG*	0.158	0.002	***	0.591	0.021	*	0.338	0.008	**
Δ*metJ*	0.163	0.000	***	0.499	0.000	***	0.249	0.000	***
Δ*baeS*	0.267	0.003	***	0.536	0.003	***	0.434	0.002	***
Δ*fepG*	0.477	0.021	*	0.206	0.000	***	1.014	0.359	ns
Δ*dcuA*	0.530	0.037	*	0.538	0.021	*	0.773	0.100	ns
Δ*fepB*	0.556	0.023	*	0.541	0.047	*	0.803	0.045	*
Δ*ccmH*	0.556	0.039	*	0.773	0.026	*	0.755	0.203	ns
Δ*sdhC*	0.587	0.022	*	0.824	0.011	*	0.637	0.015	*
Δ*gltS*	0.679	0.010	**	0.577	0.028	*	0.615	0.020	*
Δ*narU*	0.687	0.033	*	0.695	0.013	*	0.656	0.002	**
Δ*yabL*	0.791	0.042	*	0.883	0.247	ns	0.752	0.003	**
Δ*malM*	0.819	0.223	ns	0.709	0.015	*	0.713	0.016	*
Δ*ddpD*	0.819	0.045	*	0.896	0.122	ns	0.773	0.040	*
Δ*rfa*	0.846	0.009	**	0.719	0.043	*	0.454	0.026	*
Δ*glpD*	1.004	0.980	ns	1.045	0.743	ns	0.693	0.009	**

ns indicates no significant difference, * indicates *p* < 0.05, ** indicates *p* < 0.01, and *** indicates *p* < 0.001.

It should be noted that there are several reported virulence-related factors of porcine ExPEC that have not been identified in our TraDIS assay, such as the twin-arginine translocation system [31] and the type VI secretion system (T6SS) [93]. One reason could be that our Tn library did not cover all the genes. For example, the genes encoding the T6SS were without any Tn insertions (Table S4). Also, it has been reported that TraDIS analysis using such a large mixed Tn library with an animal infection model may have experimental bottlenecks which results in inaccurate assignments of candidate fitness genes [94].

In conclusion, by TraDIS analysis with a Tn library covering insertions in 72% of the genome-encoded genes of a virulent and multi-drug resistant porcine ExPEC strain PCN033 using a mouse infection model, we identified 64 genes that may be involved in *in vivo* colonization. Genes involved in fimbriae and LPS biosynthesis, metabolism, and transcription regulation are further verified to be critical for porcine ExPEC to establish systemic infection. Our genome-wide screening for *in vivo* fitness factors will be important for understanding the pathogenesis of porcine ExPEC.

## Materials and methods

### Strains and plasmids constructions

The strains and plasmids used in this study were listed in Table S1. The primer sequences used in this study were listed in Table S2. ExPEC PCN033 strain is a multi-drug resistant clinical isolate that showed high virulence in pigs as well as in mice [27,39]. *E. coli* DH5α was used as the host strain for regular cloning. *E. coli* DH5α *pir* was used as the host strain for the propagation of pRE112 derived plasmids [95]. *E. coli* χ7213 is a diaminopimelic acid autotrophic *E. coli* strain used for transconjugation [96]. The recombinant plasmids were constructed by using a seamless cloning method with the ClonExpress MultiS One Step Cloning Kit (Vazyme, China) as previously described [31]. The gene deletion strains were constructed as previously described [31]. Briefly, the suicide plasmid containing the upstream and downstream flanking region of the target gene was delivered into ExPEC PCN033 strain via transconjugation using *E. coli* χ7213 strain as the donor strain. The single exchange strain was obtained and verified by PCR which was then screened on lysogeny broth (LB) agar plate containing 10% sucrose for the double exchanged mutant.

### Construction of transposon mutagenesis library

Transposon mutagenesis in ExPEC PCN033 strain was achieved using the transposon-containing suicide plasmid pSAM-Tat-Apra (Fig. S1A) delivered by transconjugation. Briefly, the auxotrophic *E. coli* χ7213 strain (nalidixic acid and chloramphenicol sensitive) carrying pSAM-Tat-Apra plasmid was used as the donor strain and ExPEC PCN033 as the recipient (nalidixic acid resistant and chloramphenicol sensitive). The donor cells and the recipient cells were grown to the mid-log phase, washed three times with sterile saline, and mixed with a ratio of 1:3. The mixture was dropped onto a sterile filter membrane and placed on pre-warmed LB agar plate containing 2, 6-diaminopimelic acid (DAP) (final concentration 50 μg/ml). After incubation for 3 h at 37°C, the filter membrane was taken and the bacteria were washed off with LB and plated on LB agar plate containing nalidixic acid (final concentration 20 μg/ml) and chloramphenicol (final concentration 50 μg/ml) followed by incubation overnight at 37°C. It was found that after transconjugation the colonies that grew on the chloramphenicol-containing LB plates were not all transposon mutants but also contained some donor cells although they should not grow. Therefore, a second-round screening was performed by patching individual colonies to chloramphenicol-containing LB plates followed by incubation at 37°C overnight. The colonies that can grow again on the chloramphenicol-containing plates were further verified by PCR amplification with primer pair kpsm-F/R which targets the *kpsM* gene, a specific gene present in PCN033 strain but absent in χ7213 strain, Transposon-Cmr-F/R which targets the chloramphenicol resistance cassette, and R6K-F/R which target the backbone of pSAM-Tat-Apra plasmid. The transposon mutant library was generated by mixing the individual transposon mutants from different batches which was then stored at −80°C until use.

### Experimental animals and ethics statement

The specific pathogen-free (SPF) Kunming mice were purchased from the Experimental Animal Center, Huazhong Agricultural University, Wuhan, China. All animal experiments were approved by the Laboratory Animal Monitoring Committee of Huazhong Agricultural University and performed according to the recommendations in the Guide for the Care and Use of Laboratory Animals of Hubei Province, China (approval number HZAUMO-2021–0095).

### Animal infection with transposon library

The transposon mutant library stored at −80°C was reactivated by two-time consecutive passages in LB at 37°C. Then, the bacterial culture was grown to the mid-log phase, washed twice with sterile saline, and 100 μl of the bacterial suspension containing 5.6 × 10^7^ CFU of cells was taken as the input library to intraperitoneally inject each mouse. At 12 h post-infection (hpi), the mice were euthanized, and the brain, spleen, and lung tissues were taken, homogenized, diluted, and plated on chloramphenicol-containing plates. After overnight incubation at 37°C, the colonies were scraped out from the plates, serving as the output libraries (output_brain_, output_spleen_, output_lung_, respectively). A total of 9 mice were used in the above infection assay with bacteria collected from 3 of the mice were regarded as a replicate.

### *In vitro* cell and invasion adhesion assay and macrophage phagocytosis assay

Baby hamster kidney cells (BHK-21) BHK-21, and murine monocyte/macrophage cell line RAW 264.7 cells were cultured in Dulbecco’s modified Eagle’s medium supplemented with 10% heat-inactivated fetal bovine serum in a 37°C incubator with 5% CO_2_. To do *in vitro* cell adhesion and invasion assay, bacterial cells were subcultured in LB from overnight-grown culture and grown at 37°C to the mid-log phase. The bacterial cells were washed twice with sterile saline and mixed with BHK-21 cells with a multiplicity of infection (MOI) of 10:1 in a six-well plate followed by incubation at 37°C for 1 h. The cell culture was then washed twice with sterile PBS and sterile water was then added which was incubated at 4°C for 1 h for cell lysis. The lysate was serially diluted and applied to LB agar plates for bacterial counting. For macrophage phagocytosis, the bacterial cells were mixed with RAW264.7 cells with a multiplicity of infection (MOI) of 10:1 followed by incubation at 37°C for 1 h. Then, the cells were washed twice with sterile PBS, and the unphagocytized bacterial cells were killed by incubation with PBS containing chloramphenicol (final concentration 50 μg/ml). The macrophage cells were then lysed with sterile water and the lysate was serially diluted and applied to LB agar plates for bacterial counting.

### Serum killing assay

Bacterial cells were subcultured in LB from overnight-grown culture and grown at 37 Bacterial cells were subcultured in LB from °C to the mid-log phase. Cells were washed with sterile saline and 5 × 10^5^ CFU of the cells were incubated with 75% fresh or heat-inactivated serum for 20 min at 37°C. Afterward, the mixture was serially diluted and plated on LB agar plates for viable cell enumeration.

### Transposon library sequencing

The insertion site of transposon on the chromosome of an individual mutant was determined using an arbitrary PCR method as previously described [97]. In brief, the first round of PCR was performed using the genomic DNA of the transposon mutant as the template with the abridged primer pair CmR-in-1 and Adapter primer1, then the PCR product was used as a template for the second round of amplification using primer pair CmR-in-2 and Adapter primer 2. The PCR product was then sequenced to accurately identify the insertion site on the chromosome. The Tn insertion sites of the Tn library were determined by enriching the chromosomal regions flanking the transposon followed by high throughput sequencing. Briefly, bacterial genomic DNA was extracted from the input and the output libraries using E.Z.N.A.® Bacterial DNA Kit (Omega Bio-Tek, USA). Then, TIANSeq Fragment/Repair/Tailing Module kit (Cat# NG301, TIANGEN Biotech, China) was used for DNA fragmentation, end repair, and 3’ end dA-tailing. The Illumina universal adaptors were added using the TIANSeq Quick Connect Module (Cat# NG303, TIANGEN Biotech, China) which was then purified using Magic DNA Select Beads (Cat# M3022, Magic Bio, China). Primer pair F-/R- was used in the first round PCR amplification for the enrichment of the DNA region flanking the transposon. The PCR product was purified and used as the template for the second round amplification using P5/7 primer pair, and the amplification product was purified which was used to construct Illumina sequencing library by using Magic DNA Select Beads (Cat# M3022, Magic Bio, China). The DNA library was sequenced using an Illumina Novaseq sequencer. Sequence data have been submitted to NCBI GEO database with the accession number GSE197084 (The data are accessible via the link: https://www.ncbi.nlm.nih.gov/geo/query/acc.cgi?acc=GSE197084 by using the secure token: sbobaoowhdynjor).

### TraDIS data analysis

The above obtained raw sequencing reads were subjected to process by using Trimmomatic to remove sequencing adapter sequences and low-quality reads [98]. The resulting clean reads were aligned to the ExPEC PCN033 (GenBank: CP006632.1) genome using BWA (mem algorithm) [99]. The resulting bam files were sorted by using Sambamba (default parameter) to exclude redundant sequences [100]. Correlation coefficients between duplicates within groups were calculated using the StandardScaler submodule of the sklearn module of the python programming language. DEseq2 was used to compare the change in the total number of insertion reads for each gene between the input and output libraries [101]. Fold-change (FC) > 2 and *p* value <0.05 was used as the threshold to identify differentially enriched genes.

### Growth assay

Cells of the indicated strain were subcultured from overnight-grown cultures into LB, M9 medium, or M9 medium supplemented with the additives in a 96-well plate to give an initial OD _600 nm_ value of 0.01. The growth was monitored using an automatic plate reader (Bioscreen C, FP-1100-C, Oy Growth Curves AB, USA) at 37°C with shaking. Five replicates were tested for each strain.

### Competitive infection assay

A competitive infection assay was used to compare the *in vivo* fitness of each mutant strain and the wild-type PCN033 strain (WT) as described previously [31]. Briefly, the overnight-grown cultures of the mutant strain and WT strain were subcultured into LB and grown to the mid-log phase at 37°C with shaking. The two strains to be tested were mixed with a ratio of 1:1 which contained a total of 6 × 10^5^ CFU of cells. The bacterial suspension was used to intraperitoneally inject mice with five mice in each group. At 12 hpi, mice were euthanized, and the brain, spleen, and lung tissues were taken, homogenized, diluted, and plated on LB agar plates with and without appropriate antibiotics, respectively, to distinguish the mutant and the wild-type cells. The bacteria were enumerated and the competition index (CI) was calculated as the mutant-to-WT ratio within the recovered sample divided by the corresponding ratio in the inoculum.

### Mouse survival assay

ExPEC strains were subcultured from overnight-grown cultures into LB and grown to the mid-log phase at 37°C with shaking. The cells were harvested by centrifugation, washed with sterile saline, and diluted to appropriate concentrations with saline. 0.2 ml of the bacteria suspension containing approximately 6.9 × 10^5^ CFU was used to infect 4-week-old Kunming mice intraperitoneally with 6 mice in each group. Serial dilutions of each inoculum were plated to confirm the dose. The survival of mice was recorded. Statistical differences in survival between the mutant group and the WT group were determined using the log-rank test.

### Statistical analysis

Statistical analysis was performed using GraphPad Prism (version 8) software. The Student’s t-test (unpaired, two-tail) was used to calculate the statistical difference between the two groups as well as the statistical difference of the competitive indices. Significant differences are indicated by “*”*“*,” where *p* > 0.05, ns; 0.05 > *p* > 0.01, *; 0.01 > *p* > 0.001, **; *p* < 0.001, ***. Error bars in the graphs represent the standard deviations of the means.

## Supplementary Material

Supplemental Material

## Data Availability

The Tn-seq data are accessible via the link: https://www.ncbi.nlm.nih.gov/geo/query/acc.cgi?acc=GSE197084 by the using secure token: sbobaoowhdynjor.
