# Supplementary material for: Genome-wide identification of genes critical for in vivo fitness of multi-drug resistant porcine extraintestinal pathogenic Escherichia coli by transposon-directed insertion site sequencing using a mouse infection model
Source: Virulence. 2023 Jan 4;14(1):2158708. doi: 10.1080/21505594.2022.2158708 (PMC9828833; doi:10.1080/21505594.2022.2158708)
Supplement: Supplemental Material [file KVIR_A_2158708_SM2854.zip › supplementary/Table S1 Strains and plasmids-R3.docx]

**Table S1. Strains and plasmids used in this study**

| **Name** | **Description** | **Source** |
| --- | --- | --- |
| Strains |  |  |
| ExPEC PCN033 | A virulent multi-drug resistant clinical strain isolated from a diseased pig. Cm^S^, Apr^S^, Nal^R^, and Amp^R^. | (Tan et al., 2011) |
| *E. coli* DH5α | Host strain for regular cloning. | CW Bio Co., Ltd. |
| *E. coli* DH5α *pir* | Host strain for the propagation of pRE112 derived plasmids. | (Platt et al., 2000) |
| *E. coli* χ7213 | A diaminopimelic acid autotrophic *E. coli* strain used for transconjugation. Nal^S^. | (Roland et al., 1999) |
| Δ*fepB* | As *E. coli* PCN033, *fepB* deleted. | This study |
| Δ*fepG* | As *E. coli* PCN033, *fepG* deleted. | This study |
| Δ*fimG* | As *E. coli* PCN033, *fimG* deleted. | This study |
| Δ*ddpD* | As *E. coli* PCN033, *ddpD* replaced with apramycin resistance cassette. Apr^R^. | This study |
| Δ*yabL* | As *E. coli* PCN033, *yabL* replaced with apramycin resistance cassette. Apr^R^. | This study |
| Δ*narU* | As *E. coli* PCN033, *narU* replaced with apramycin resistance cassette. Apr^R^. | This study |
| Δ*malM* | As *E. coli* PCN033, *malM* replaced with apramycin resistance cassette. Apr^R^. | This study |
| Δ*dcuA* | As *E. coli* PCN033, *dcuA* replaced with apramycin resistance cassette. Apr^R^. | This study |
| Δ*ccmH* | As *E. coli* PCN033, *ccmH* replaced with apramycin resistance cassette. Apr^R^. | This study |
| Δ*sdhC* | As *E. coli* PCN033, *sdhC* replaced with apramycin resistance cassette. Apr^R^. | This study |
| Δ*glpD* | As *E. coli* PCN033, *glpD* replaced with apramycin resistance cassette. Apr^R^. | This study |
| Δ*baeS* | As *E. coli* PCN033, *baeS* replaced with apramycin resistance cassette. Apr^R^. | This study |
| Δ*ccdB* | As *E. coli* PCN033, *ccdB* replaced with apramycin resistance cassette. Apr^R^. | This study |
| Δ*rfa* | As *E. coli* PCN033, *rfa* replaced with apramycin resistance cassette. Apr^R^. | This study |
| Δ*metJ* | As *E. coli* PCN033, *metJ* replaced with apramycin resistance cassette. Apr^R^. | This study |
| Δ*gltS* | As *E. coli* PCN033, *gltS* replaced with apramycin resistance cassette. Apr^R^. | This study |
| PCN033-Chl | As *E. coli* PCN033, a chloramphenicol resistance cassette inserted at an intergenic region. Cm^R^. | This study |
| Plasmids |  |  |
| pRE112 | The plasmid used for mutant construction. | (Edwards et al., 1998) |
| pSAM-Tat-AprR | The plasmid used for transposon mutagenesis. Cm^R^ and Apr^R^. | This study |
| pRE112-*fepB* | pRE112 containing 1000 bp sequence upstream *fepB* followed by 1000 bp downstream *fepB.* Cm^R^. | This study |
| pRE112-*fepG* | pRE112 containing 1000 bp sequence upstream *fepG* followed by 1000 bp downstream *fepG.* Cm^R^. | This study |
| pRE112-*fimG* | pRE112 containing 1000 bp sequence upstream *fimG* followed by 1000 bp downstream *fimG.* Cm^R^. | This study |
| pRE112-*ddpD* | pRE112 containing a apramycin sulfate resistance cassette flanked by 1000 bp upstream and downstream of *ddpD.* Cm^R^ and Apr^R^. | This study |
| pRE112-*yabL* | pRE112 containing a apramycin sulfate resistance cassette flanked by 1000 bp upstream and downstream of *yabL.* Cm^R^ and Apr^R^. | This study |
| pRE112-*narU* | pRE112 containing a apramycin sulfate resistance cassette flanked by 1000 bp upstream and downstream of *narU.* Cm^R^ and Apr^R^. | This study |
| pRE112-*malM* | pRE112 containing a apramycin sulfate resistance cassette flanked by 1000 bp upstream and downstream of *malM.* Cm^R^ and Apr^R^. | This study |
| pRE112-*dcuA* | pRE112 containing a apramycin sulfate resistance cassette flanked by 1000 bp upstream and downstream of *dcuA.* Cm^R^ and Apr^R^. | This study |
| pRE112-*ccmH* | pRE112 containing a apramycin sulfate resistance cassette flanked by 1000 bp upstream and downstream of *ccmH.* Cm^R^ and Apr^R^. | This study |
| pRE112-*sdhC* | pRE112 containing a apramycin sulfate resistance cassette flanked by 1000 bp upstream and downstream of *sdhC.* Cm^R^ and Apr^R^. | This study |
| pRE112-*glpD* | pRE112 containing a apramycin sulfate resistance cassette flanked by 1000 bp upstream and downstream of *glpD.* Cm^R^ and Apr^R^. | This study |
| pRE112-*baeS* | pRE112 containing a apramycin sulfate resistance cassette flanked by 1000 bp upstream and downstream of *baeS.* Cm^R^ and Apr^R^ | This study |
| pRE112-*ccdB* | pRE112 containing a apramycin sulfate resistance cassette flanked by 1000 bp upstream and downstream of *ccdB.* Cm^R^ and Apr^R^. | This study |
| pRE112-*rfa* | pRE112 containing a apramycin sulfate resistance cassette flanked by 1000 bp upstream and downstream of *rfa.* Cm^R^ and Apr^R^. | This study |
| pRE112-*metJ* | pRE112 containing a apramycin sulfate resistance cassette flanked by 1000 bp upstream and downstream of *metJ.* Cm^R^ and Apr^R^. | This study |
| pRE112-*gltS* | pRE112 containing a apramycin sulfate resistance cassette flanked by 1000 bp upstream and downstream of *gltS.* Cm^R^ and Apr^R^. | This study |
| pRE112-Chl | pRE112 containing a chloramphenicol resistance cassette flanked by 1000 bp upstream and downstream of the insertion site (genome position 3263584). Cm^R^. | This study |

Note: Nal^S^, nalidixic acid sensitive; Nal^R^, nalidixic acid resistant; Cm^S^, chloramphenicol sensitive; Cm^R^, chloramphenicol resistant; Apr^S^, apramycin sensitive; Apr^R^, apramycin resistant; Amp^R^

Edwards, R.A., Keller, L.H., Schifferli, D.M., 1998. Improved allelic exchange vectors and their use to analyze 987P fimbria gene expression. Gene 207, 149-157.

Platt, R., Drescher, C., Park, S.K., Phillips, G.J., 2000. Genetic system for reversible integration of DNA constructs and lacZ gene fusions into the *Escherichia coli* chromosome. Plasmid 43, 12-23.

Roland, K., Curtiss, R., 3rd, Sizemore, D., 1999. Construction and evaluation of a delta cya delta crp *Salmonella typhimurium* strain expressing avian pathogenic *Escherichia coli* O78 LPS as a vaccine to prevent airsacculitis in chickens. Avian Dis 43, 429-441.

Tan, C., Xu, Z., Zheng, H., Liu, W., Tang, X., Shou, J., Wu, B., Wang, S., Zhao, G.P., Chen, H., 2011. Genome sequence of a porcine extraintestinal pathogenic *Escherichia coli* strain. J Bacteriol 193, 5038.
