# Supplementary material for: Genome-wide identification of genes critical for in vivo fitness of multi-drug resistant porcine extraintestinal pathogenic Escherichia coli by transposon-directed insertion site sequencing using a mouse infection model
Source: Virulence. 2023 Jan 4;14(1):2158708. doi: 10.1080/21505594.2022.2158708 (PMC9828833; doi:10.1080/21505594.2022.2158708)
Supplement: Supplemental Material [file KVIR_A_2158708_SM2854.zip › supplementary/Table S2 Primers.docx]

**Table S2. Primers used in this study**

| Primer name | Sequence (5'-3') |
| --- | --- |
| Transposon-Cmr-F | CACGTAAGAGGTTCCCAC |
| Transposon-Cmr-R | CATTCTGCCGACATGGAAG |
| kpsm-F | ATGGCAAGAAGTGGATTTGAA |
| kpsm-R | CATGACGTCAGCATTGCCTC |
| R6K-F | GCCGTTAAGTGTTCCTGTG |
| R6K-R | CTGAAGATCAGCAGTTCAACC |
| CmR-in-1 | CGACGATTTCCGGCAGTTTC |
| CmR-in-2 | CGCAAGATGTGGCGTGTTAC |
| Apra-F | TCATCGGTCAGCTTCTCAAC |
| Apra-R | GCATCGCATTCTTCGCATC |
| Adapterprimer1 | GGCCACGCGTCGACTAGTACNNNNNNNNNNGATAT |
| Adapterprimer2 | GGCCACGCGTCGACTAGTAC |
| Chl-UP-R | GAACCTCTTACGTGCCGATCAGGCGACCCGTTTTTCTTTGC |
| Chl-F | GCAAAGAAAAACGGGTCGCCTGATCGGCACGTAAGAGGTTC |
| Chl-R | GATAATAATGGTTTCTTAGACGTCTTTAGCTTCCTTAGCTCCTG |
| Chl-down-F | GGCAGGGCGGGGCGTAAAGAAGGTGACCCGTTTTTTTTCTTAC |
| pRE112-F | CACTGTTCGTCCATTTCCGC |
| pRE112-R | TATTGGTGCCCTTAAACGCC |
| F- | ACACTCTTTCCCTACACGACGCTCTTCCGATCT |
| R- | GTGACTGGAGTTCAGACGTGTGCTCTTCCGATCTGCCTTTTTGCGTTTCTACCTG |
| P5primer | AATGATACGGCGACCACCGAGATCTACACTCTTTCCCTACACGACGCTCTTCCGATC*T |
| P7primer | CAAGCAGAAGACGGCATACGAGATGTGACTGGAGTTCAGACGTGTGCTCTTCCGATCT |
| ccdB-up-F | AACTGCATGAATTCCCGGGAGAGCTCCAATGATCAGTCTGATTGCG |
| ccdB-up-R | ATGGAGCTGCACATGAACCCCGCTAGAAGCTCCGGTACTC |
| ccdB-Apra-F | GAGTACCGGAGCTTCTAGCGGGGTTCATGTGCAGCTCCAT |
| ccdB-Apra-R | GAGAGGCGGTCGCGTCTTAATCATGAGCTCAGCCACTGGC |
| ccdB-down-F | GCCAGTGGCTGAGCTCATGATTAAGACGCGACCGCCTCTC |
| ccdB-down-R | GATCCCAAGCTTCTTCTAGAGGTACCTGAACTGATCGCATTGCTGC |
| fepG-up-F | AACTGCATGAATTCCCGGGAGAGCTCGCACCATCGTGCTCGACG |
| fepG-up-R | GCGTCGGGCAATGTGCGGTCATGCGCCACCTCGCGTTTTAC |
| fepG-down-F | GTAAAACGCGAGGTGGCGCATGACCGCACATTGCCCGACGC |
| fepG-down-R | GATCCCAAGCTTCTTCTAGAGGTACCGATTAAGATCGTGCAGCACC |
| fepB-up-F | AACTGCATGAATTCCCGGGAGAGCTCCATCAGCCAGCGGGACATG |
| fepB-up-R | CAGCGTCCGACAGTTAATGCGCAAAAGTCCTGTTAATAGAAG |
| fepB-down-F | CTTCTATTAACAGGACTTTTGCGCATTAACTGTCGGACGCTG |
| fepB-down-R | GATCCCAAGCTTCTTCTAGAGGTACCCGATCACCATGTTGACCGTG |
| rfa-up-F | AACTGCATGAATTCCCGGGAGAGCTCCGTTAACGCAATCTTCATTG |
| rfa-up-R | GATTTTATTTCACATATTCAATTTCTCTCCGGAAATTACA |
| rfa-down-F | TGTAATTTCCGGAGAGAAATTGAATATGTGAAATAAAATC |
| rfa-down-R | GATCCCAAGCTTCTTCTAGAGGTACCGCACAGATTCCTTCCTGGCA |
| fimG-up-F | AACTGCATGAATTCCCGGGAGAGCTCACGTCGATTTAGATAACGCG |
| fimG-up-R | GCCTGAATGGTTCCCTGAGTGAGCATCTCCAGTTACTGAT |
| fimG-down-F | ATCAGTAACTGGAGATGCTCACTCAGGGAACCATTCAGGC |
| fimG-down-R | GATCCCAAGCTTCTTCTAGAGGTACCCCTAACGATACCGTGTTATT |
| narU-up-F | AACTGCATGAATTCCCGGGAGAGCTCAGACATTAAACGCTGCGAAC |
| narU-up-R | ATGGAGCTGCACATGAACCCATTGTTCCTCACATGTACAC |
| narU-Apra-F | GTGTACATGTGAGGAACAATGGGTTCATGTGCAGCTCCAT |
| narU-Apra-R | TTAAAAACACTTTCATCGCGTCATGAGCTCAGCCACTGGC |
| narU-down-F | GCCAGTGGCTGAGCTCATGACGCGATGAAAGTGTTTTTAA |
| narU-down-R | GATCCCAAGCTTCTTCTAGAGGTACCCCAGTCGTAGAAGCTTAAAC |
| ybaL-up-F | AACTGCATGAATTCCCGGGAGAGCTCCAGCACCGAATGAATAAAGG |
| ybaL-up-R | ATGGAGCTGCACATGAACCCTCCGTCTCCTTTTCCTGGTG |
| ybaL-Apra-F | CACCAGGAAAAGGAGACGGAGGGTTCATGTGCAGCTCCAT |
| ybaL-Apra-R | TTACATCCGGCAACCACGGTTCATGAGCTCAGCCACTGGC |
| ybaL-down-F | GCCAGTGGCTGAGCTCATGAACCGTGGTTGCCGGATGTAA |
| ybaL-down-R | GATCCCAAGCTTCTTCTAGAGGTACCACTGGTTCTCACTTCTTATC |
| dcuA-up-F | AACTGCATGAATTCCCGGGAGAGCTCAAACTGGCTGAAGTCACTGG |
| dcuA-up-R | ATGGAGCTGCACATGAACCCATTAGCCTTCCTTGTTTTTT |
| dcuA-down-F | GCCAGTGGCTGAGCTCATGACTACCCGTATCGGTAAATTC |
| dcuA-down-R | GATCCCAAGCTTCTTCTAGAGGTACCACTCATCTTCATGCCAGACG |
| dcuA-Apra-F | AAAAAACAAGGAAGGCTAAT GGGTTCATGTGCAGCTCCAT |
| dcuA-Apra-R | GAATTTACCGATACGGGTAG TCATGAGCTCAGCCACTGGC |
| glpD-up-F | AACTGCATGAATTCCCGGGAGAGCTCGGATAACGGCGATAATGCAG |
| glpD-up-R | ATGGAGCTGCACATGAACCCGCTGCCCTCATTCACTTTCG |
| glpD-Apra-F | CGAAAGTGAATGAGGGCAGCGGGTTCATGTGCAGCTCCAT |
| glpD-Apra-R | CCTGACCACCTTACGTTAAATCATGAGCTCAGCCACTGGC |
| glpD-down-F | GCCAGTGGCTGAGCTCATGATTTAACGTAAGGTGGTCAGG |
| glpD-down-R | GATCCCAAGCTTCTTCTAGAGGTACCTGATTCGCTGATCAACTTCG |
| gltS-up-F | AACTGCATGAATTCCCGGGAGAGCTCCAACCTGAATTAGCGACAGG |
| gltS-up-R | ATGGAGCTGCACATGAACCCAGTTACTCCTTTGTATCCGC |
| gltS-Apra-F | GCGGATACAAAGGAGTAACTGGGTTCATGTGCAGCTCCAT |
| gltS-Apra-R | GAAGACCGCCGCTTCATCGGTCATGAGCTCAGCCACTGGC |
| gltS-down-F | GCCAGTGGCTGAGCTCATGACCGATGAAGCGGCGGTCTTC |
| gltS-down-R | GATCCCAAGCTTCTTCTAGAGGTACCCTGCGTATGCCGATCTTGAT |
| malM-up-F | AACTGCATGAATTCCCGGGAGAGCTCAACTATCGTCTGGTTGATGG |
| malM-up-R | ATGGAGCTGCACATGAACCCTGTTTTCACCTCAAAATCTG |
| malM-Apra-F | CAGATTTTGAGGTGAAAACAGGGTTCATGTGCAGCTCCAT |
| malM-Apra-R | ATCAGCACTGTGGGGCGTAATCATGAGCTCAGCCACTGGC |
| malM-down-F | GCCAGTGGCTGAGCTCATGATTACGCCCCACAGTGCTGAT |
| malM-down-R | GATCCCAAGCTTCTTCTAGAGGTACCAGTTGACTCTGCGAGAAGTC |
| sdhC-up-F | AACTGCATGAATTCCCGGGAGAGCTCGAACCGAGAGTACGGATATC |
| sdhC-up-R | ATGGAGCTGCACATGAACCCGAATAACGCCCACATGCTGT |
| sdhC-Apra-F | ACAGCATGTGGGCGTTATTCGGGTTCATGTGCAGCTCCAT |
| sdhC-Apra-R | CAAAGGAGATTTTGGCGGAGTCATGAGCTCAGCCACTGGC |
| sdhC-down-F | GCCAGTGGCTGAGCTCATGACTCCGCCAAAATCTCCTTTG |
| sdhC-down-R | GATCCCAAGCTTCTTCTAGAGGTACCGTTCTGCTGATAAAGCGTGT |
| ddpD-up-F | AACTGCATGAATTCCCGGGAGAGCTCCCGTGCAAAACTGGTATGGA |
| ddpD-up-R | ATGGAGCTGCACATGAACCCTCATGACTGCTTTCCTCCTG |
| ddpD-Apra-F | CAGGAGGAAAGCAGTCATGAGGGTTCATGTGCAGCTCCAT |
| ddpD-Apra-R | CGGTATTCCATGTTCCGGTTCATGAGCTCAGCCACTGGC |
| ddpD-down-F | GCCAGTGGCTGAGCTCATGAACCGGAACATGGAATACCGC |
| ddpD-down-R | GATCCCAAGCTTCTTCTAGAGGTACCCATAAGTCAGCTCGCGATTT |
| baeS-up-F | AACTGCATGAATTCCCGGGAGAGCTCTGCCGAACTACACCATGCAG |
| baeS-up-R | ATGGAGCTGCACATGAACCCTCATTGCGCGCTCCTTTTTC |
| baeS-Apra-F | GAAAAAGGAGCGCGCAATGAGGGTTCATGTGCAGCTCCAT |
| baeS-Apra-R | AATTTTTGTAGCTGATCGTCTCATGAGCTCAGCCACTGGC |
| baeS-down-F | GCCAGTGGCTGAGCTCATGAGACGATCAGCTACAAAAATT |
| baeS-down-R | GATCCCAAGCTTCTTCTAGAGGTACCCACGGATAAAGGACTGTTCG |
| ccmH-up-F | AACTGCATGAATTCCCGGGAGAGCTCACACTGCTGGGTCGATGATG |
| ccmH-up-R | ATGGAGCTGCACATGAACCCTCATTGTGCGGCCTCCTTAC |
| ccmH-Apra-F | GTAAGGAGGCCGCACAATGAGGGTTCATGTGCAGCTCCAT |
| ccmH-Apra-R | CATCACCATCGGGCCTCTTATCATGAGCTCAGCCACTGGC |
| ccmH-down-F | GCCAGTGGCTGAGCTCATGATAAGAGGCCCGATGGTGATG |
| ccmH-down-R | GATCCCAAGCTTCTTCTAGAGGTACCCAAAGCGAACGACAAGGTTG |
| metJ-up-F | AACTGCATGAATTCCCGGGAGAGCTCCGTAGCAGCAGATAGCTGTC |
| metJ-up-R | ATGGAGCTGCACATGAACCCGAGATACTTAATCCTCTTCG |
| metJ-Apra-F | CGAAGAGGATTAAGTATCTCGGGTTCATGTGCAGCTCCAT |
| metJ-Apra-R | CCGCGCCGCTCTTTTCCGCTTCATGAGCTCAGCCACTGGC |
| metJ-down-F | GCCAGTGGCTGAGCTCATGAAGCGGAAAAGAGCGGCGCGG |
| metJ-down-R | GATCCCAAGCTTCTTCTAGAGGTACCGAAGAAATTACTGCTAGCTG |
| fepG-out-F | AGCACGCACACAGTTGATTG |
| fepG-out-R： | AGCTCCTGCACGGTGATATC |
| fepG-in-F | GCGTGCTGATGGCGCTGTTG |
| fepG-in-R | GCAACCAGCATCATTAACAG |
| fepB-out-F | GCGAAACATCCTGACGTCG |
| fepB-out-R | GATTTGTCTGGCGCTATTCG |
| fepB-in-F | AACGCAAACTGCAACGGCTC |
| fepB-in-R | CCAGCGCATAAACCTGCTTG |
| rfa-out-F | TCGTTGGCGCGATAAAGATC |
| rfa-out-R | AAGACCTGCACTTCTGACAC |
| rfa-in-F | GAACGCGATGCTAACTGGTG |
| rfa-in-R | GGCATCCAGATACAGCAGTG |
| fimG-out-F | CGTTTCTGCCGTAAAAGTTG |
| fimG-out-R | AATGGATAGCTACTGCCAC |
| fimG-in-F | AACGTGGGTATTTATTGGCG |
| fimG-in-R | GAGGAATCATCCACCTGAAC |
| sdhC-out-F | GACCTGGATCACTGTTCAGG |
| sdhC-out-R | GTGCAACGACAATCACCAG |
| sdhC-in-F | AAGACCTGTTAATCTGGAC |
| sdhC-in-R | GCTTCGAATGTTTCTTCCAG |
| ddpD-out-F | ATTGTGCAGGCATCGCTG |
| ddpD-out-R | GCATAATGCGTTGTGATCC |
| ddpD-in-F | CGCTCAACAATGTGTCCTTG |
| ddpD-in-R | GTGCGCATTGCAGCAAAC |
| baeS-out-F | GCAATTGTCGATGAGTATCG |
| baeS-out-R | CGTCGATTGGTAACTCGGTC |
| baeS-in-F | TTGCTGATCAGTATGCACTG |
| baeS-in-R | CGAGCTGGTTGAAGTCTTGC |
| ccmH-out-F | CGGCATGAACTATAAAGACG |
| ccmH-out-R | GAGCAGAAGAAGATCCCTTC |
| ccmH-in-F | GTTGCAGTTTAAGGATGAAG |
| ccmH-in-R | TGTCAACGCTTCAGCGTATC |
| glpD-out-F | CTCTGTGGATCGCGAATATC |
| glpD-out-R | GAGTGGGTAATGTGTCTGAG |
| glpD-in-F | TACGATCATCTGGGTAAACG |
| glpD-in-R | TAGGTCCAGACGATATCATC |
| gltS-out-F | AATCCCGTGGTAGCAAAGC |
| gltS-out-R | AGACTTAGAGATTCGCGGAC |
| gltS-in-F | ATACACCATACCGGAACCAG |
| gltS-in-R | ACAGTACACAGACGAAGGTC |
| malM-out-F | AACACTAACTTCGGCAAAGC |
| malM-out-R | CCAGAATTTGTGATATGACC |
| malM-in-F | CAATCTAAAACCCAGACCAC |
| malM-in-R | AACTTTCCGTGTCGTTGAGC |
| dcuA-out-F | GTAAGAGTGTACGTGAAGTC |
| dcuA-out-R | CATCAAGCATAAACACCTCG |
| dcuA-in-F | CATTATCATGGCGGTTATCG |
| dcuA-in-R | GATGATCAGGATTGCGTTGG |
| narU-out-F | GGGTTGAAACATGACATACG |
| narU-out-R | TGAAGTAGCGAAAGCGATCC |
| narU-in-F | CGTTATCTTTTACGCGACTG |
| narU-in-R | AGCTGCATCACACTTACACC |
| ybaL-out-F | GATTTCATTGCAGCCAAAGG |
| ybaL-out-R | TGAGCTATCAGGTACTGAAC |
| ybaL-in-F | GTCGTTAATGACCGGTATCG |
| ybaL-in-R | GCTAACGACTTACCAAACAG |
| metJ-out-F | TCGGTGAAATGTCAGGCACC |
| metJ-out-R | ATCACCAGCATACCGGTGTG |
| metJ-in-F | ATGGAGCGGCGAATATATC |
| metJ-in-R | TAGTATTCCCACGTCTCC |
| ccdB-out-F | GCATCACTGATACGTGCAG |
| ccdB-out-R | ACTGCGGTTTATCACCAACG |
| ccdB-in-F | TTTACGGTATACCGCAGTCG |
| ccdB-in-R | GTCGAGCATAAAATCTAATG |
