# Supplementary figures and images for: Genome-wide identification of genes critical for in vivo fitness of multi-drug resistant porcine extraintestinal pathogenic Escherichia coli by transposon-directed insertion site sequencing using a mouse infection model
Source: Virulence. 2023 Jan 4;14(1):2158708. doi: 10.1080/21505594.2022.2158708 (PMC9828833; doi:10.1080/21505594.2022.2158708)

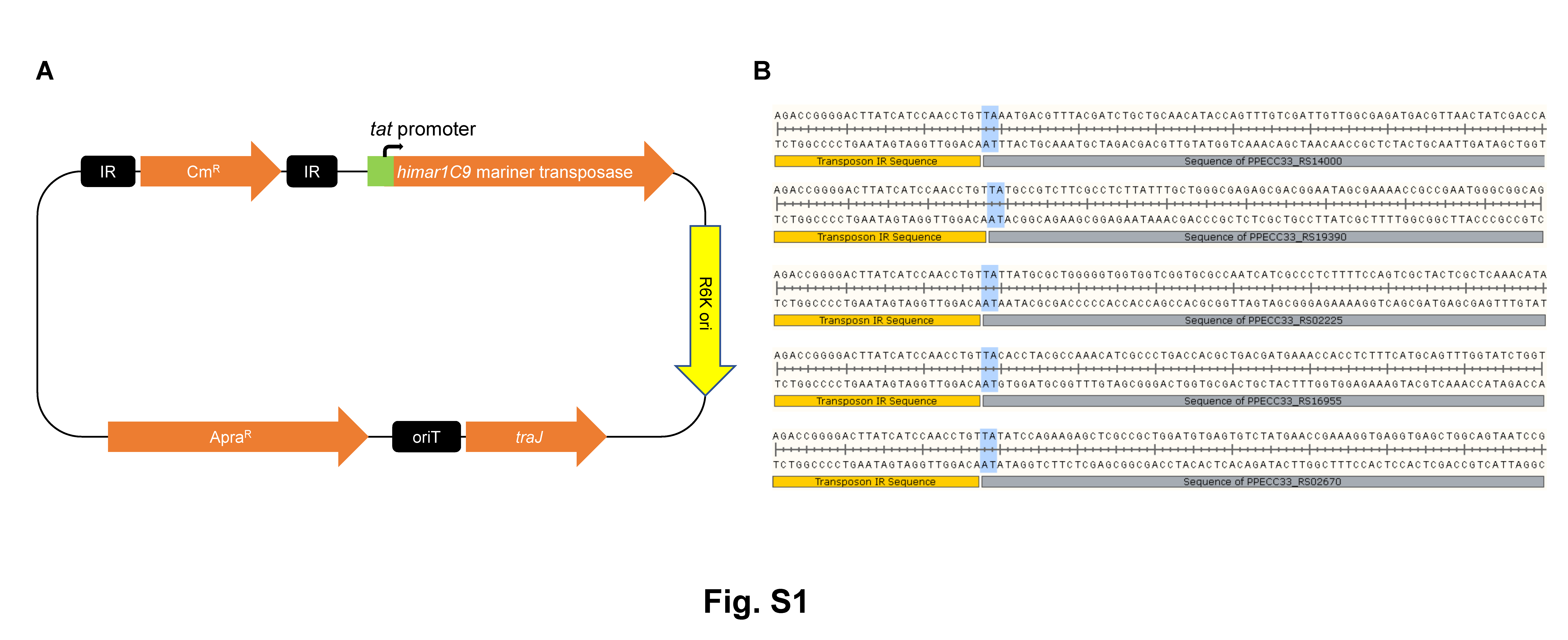

Supplement: Supplemental Material [file KVIR_A_2158708_SM2854.zip › supplementary/FigS1.tif]
